# Application of non-invasive neuromodulation in children with neurodevelopmental disorders to improve their sleep quality and constipation

**DOI:** 10.1186/s12887-023-04307-4

**Published:** 2023-09-15

**Authors:** Aníbal Báez-Suárez, Iraya Padrón-Rodríguez, Elizabeth Castellano-Moreno, Erica González-González, María P. Quintana-Montesdeoca, Raquel Irina Medina-Ramirez

**Affiliations:** 1 University of Las Palmas de Gran Canaria, Las Palmas, Spain; 2 Rehabilitation Department, Ciudad San Juan de Dios, Las Palmas, Spain

**Keywords:** Children, Constipation, Physical therapy modalities, Sleep quality, Vagus nerve stimulation

## Abstract

**Background:**

Children with neurodevelopmental disorders have a very wide clinical variability. A common prevalent factor is problems with stool and sleep quality. Currently, there are multiple studies related to their evaluation, but not so much related to a specific intervention. The aim was to evaluate the effectiveness and safety of the application of non-invasive neuromodulation as a treatment in children with neurodevelopmental disorders to improve constipation and quality of sleep.

**Methods:**

A total of 23 minors aged between 2 and 16 were included in this cross-sectional study. All participants were applied the microcurrent device for 60 min, 3 times per week for a total of 4 weeks. The technique was based on non-invasive neuromodulation using a surface-applied microcurrent electrostimulation device that administers an external, imperceptible, pulsed electrical stimulation. It is applied to the extremities, in a coordinated manner, using gloves and anklets connected with electrodes to a control console. Sleep latency and microarousals were evaluated through a sleep diary. To assess the evolution and type of defecation, the adapted and validated version in Spanish of the Bristol Stool Form Scale was used.

**Results:**

No adverse events occurred during the study and no incidences were registered. Clinically relevant improvements were registered in defecation frequency and type as well as in sleep related parameters. An increase in the hours of sleep was registered, from 7,35 (0,83) to 9,09 (1,35), and sleep interruptions decreased from 3,83 (1,95) to 1,17 (1,11), (p < .001).

**Conclusion:**

Microcurrents can be used as an effective and safe treatment to improve quality of sleep and constipation in children with neurodevelopmental disorders. More studies are needed in order to obtain statistically significant results.

**Trial Registration:**

ClinicalTrials.gov ID: NCT05265702.

**First registration:**

03/03/2022

**Protocol:**

https://clinicaltrials.gov/ct2/show/NCT05265702?term=baez+suarez&draw=2&rank=4

## Background

Neurodevelopmental disorders belong to a ‘heterogeneous group of conditions characterized by a delay or alteration in the acquisition of skills in several development areas including motor, social, language and cognition’. They are grouped in a variety of clinical disorders that cause symptoms in early stages of life, which affect development evolution in the aforesaid areas. The severity of the deterioration and the variety of symptoms associated with it, reflect dysfunctions -focal or global, structural or functional- in neural networks. [1] It affects between 15 and 20% of children, being a frequent reason for consultation during childhood and adolescence. Lack of detection, diagnosis and treatment result in underdiagnosed effects in adulthood, with loss of opportunities in developing individual potential in their personal, family and professional life. [1, 2]

The majority of these children, present a delay in the acquisition of skills according to the phases of typical psychomotor development. Added to this difficulty, and main element of concern for family members, there are another series of signs that appear with some frequency and, which despite being unnoticed over other major problems, also represent basic and fundamental factors for a correct development and performance of children, such as constipation problems and sleep disorders. [3].

Focusing on these two variables, the relationship between constipation and sleep disturbance has been studied in healthy adults, [4, 5] or adults with a pathology, mostly based on people with Parkinson’s disease. [6–8]. In adults with functional gastrointestinal disorder, inadequate sleep, delayed sleep onset, and poor sleep quality are common and contribute to increased abdominal pain and gastrointestinal symptom severity concurrently and next-day [9, 10]. At younger ages, most youth with chronic pain complain about sleep disturbances, including short sleep duration, poor quality sleep, and frequent night awakenings. However, current evidence provides little or no information on this relationship in children with neurodevelopmental impairment, with the majority focusing on incontinence problems. [11–13] Paediatric patients with neurological involvement have comorbidities at different levels of the gastrointestinal tract, which represents a complex problem requiring long-term multidisciplinary follow-up. It is therefore of great interest to be able to respond to this problem.

Sleep disorder treatment in children with neurodevelopmental disorders, frequently require not only adequate sleep hygiene measures but also pharmacological treatment with the aim of minimizing the impact of sleeping disorders in the child. [14]Therefore, there is an interest in evaluating new non-pharmacological approaches, such as non-invasive neuromodulation.

Non-invasive global neuromodulation is based on a superficial treatment, with very subtle electrical microcurrents, provoking imperceptible sensations which are very comfortable for the patient. The effect of the electrical microcurrent is multiplied, as it is delivered by multiple pathways that structurally cover the whole body through electrodes in the limbs and a directing electrode. Due to its characteristics, non-invasive neuromodulation has an enormous potential for clinical applications in the rehabilitation field.

The main objective of this study is to evaluate the effectiveness and safety of the application of non-invasive neuromodulation as a treatment in children with neurodevelopmental disorders to improve constipation and quality of sleep.

## Methods

### Study design

In this observational study, the sample was selected through a non-probability sampling by convenience, due to its characteristics, availability and type of study design. The research was conducted according to the Strengthening the Reporting of Observational Studies in Epidemiology (STROBE) guidelines.

### Population

The study population were students at *‘Centro Ciudad San Juan de Dios’* in Las Palmas de Gran Canaria, Spain, with a diagnosis associated to neurodevelopmental disorders. Data was collected between May and April 2022. The type of recruitment consisted of a non-probabilistic convenience method.

The inclusion criteria was to present a neurodevelopmental disorder (Level I to V according to the Gross Motor Function Measure, GMFM), aged between 2 and 18, to be a student at the centre of reference of the study, meet the requirements of the constipation scale of the Rome IV Criteria, and that the informed consent was signed by the family, guardian or legal representative.

### Intervention

Participants who met the inclusion and exclusion criteria, were treated with the non-invasive neuromodulation device (NESA XSignal) for 60 min, three times a week for a total of 12 sessions. The parameters applied consist of a low frequency oscillating microcurrent from 1,3 Hz to 14,28 Hz, with an intensity of 0.5 milliamperes and a potential difference of ± 3 V and ± 6 V. Impulses are fired in a coordinated manner between 24 electrodes (6 electrodes per limb) which fire simultaneously. 6 electrodes are placed in each limb, in different points of the peripheral nerve with low impedance, in the form of a set of gloves and adapted socks for hands and feet. Electrodes are connected to the main control unit and an adhesive electrode is placed on the cervical spine at C7 level. The protocol used was the application of program 2 for 15 min followed by the application of program 7 for the remaining 45 min.

During treatment time, all participants continued with their usual routine in the centre.

In order to assess and ensure safety during the intervention, an adverse event registry was kept. Participants reactions were observed during the sessions. As it is an imperceptible procedure from a sensation point of view and easily manageable, participants were allowed to conduct other activities (school/rehabilitation) simultaneously.

### Description of outcome measures

A bowel movement registry was kept. It consisted on a daily assessment of the number of bowel movements and the type of stool according to the criteria set with the Bristol Stool Form Scale (BSS). The BSS is the most used standardized tool to assess the consistency of stools in children in clinical settings and in research. According to the BSS, which classifies stools in 7 categories, types 1 and 2 are hard and indicate constipation, types 3 to 5 are considered to be the normal stool forms (being type 4 the most normal) and type 6 and 7, loose and liquid, indicate diarrhea. [15] The BSS image table was accompanied by descriptors, using the translated and validated Spanish version. [16].

A sleep diary was the tool used to calculate the number of hours of sleep, sleep latency, sleep arousal and sleep routines. The validity of the data obtained with this resource is comparable to that obtained with actigraphy. [17–19]A sleep diary is easily completed, and it provides a global vision of sleep for several days. This diary included questions about bedtime, estimated time to fall asleep (sleep onset latency) and wake-up time. Also, on how many occasions did the person wake up during the night and the duration of total time awake, between lying down and getting up, was recorded.

Data was collected by people who surround the child: carers, physiotherapists, speech and language therapists, occupational therapists and/or the classroom teacher when at the centre. Data was also collected by the family, guardian or legal representative of the place where the child was staying. To avoid bias in data collection, families were informed to start recording the data, however, neuromodulation was not applied for a week after announcing that the study had begun.

All variables were registered at an initial stage (first week without intervention), during the four weeks where non-invasive neuromodulation was applied and two weeks after the intervention.

### Ethics

Parents/legal guardians of the participants were informed of the aim of the study and informed consent was obtained in writing. The study was conducted according to the principles described in the Declaration of Helsinki. The study protocol was approved by the Human Research Ethics Committee of *‘Dr. Negrín’* University Hospital of Gran Canaria, Spain (ID: CEIm- 2022-096-1), and the study was registered at ClinicalTrials.gov on 03/03/2022. (ID: NCT05265702).

### Statistical analysis

For the statistical analysis of data, Statistical Package for the Social Sciences (SPSS) version 20.0 software was used. To obtain the data, a univariate and bivariate descriptive analysis was performed.

For the univariate analysis, qualitative variables were expressed as absolute frequencies and percentages, with an estimated 95% confidence interval. Quantitative variables were expressed as means ± standard deviation.

To compare the means between two independent samples, T-Student was used or its non-parametric equivalent of Mann-Whitney and/or Wilcoxon test, whereas in order to determine the association between qualitative variables the Chi-squared Test of Independence was used. Previously, a Kolmogorov-Smirnov Test of Normality was performed.

## Results

The sample consisted of 23 individuals whose general characteristics are summarized in the following tables. No incidences occurred that had to be registered in 100% of cases. The general characteristics of the sample, in relation with age and degree of disability, are represented in Table 1.

**Table 1:** General Characteristics

Variable		% (n)	
Sex	Male	26.1% (6)	
	Female	73.9% (17)	
Classification according to the child’s potential (GMFM)	Grade I	30.4% (7)	
	Grade II	39.1% (9)	
	Grade III	17.4% (4)	
	Grade IV	4.3% (1)	
	Grade V	8.7% (2)	
Age (years)	**Media (DT)**	**P50 (IQR)**	**Min. – Max.**
	6.78 (4.26)	6 (4)	2–16

SD: Standard Deviation; IQR: Interquartile Range (P75-P25)

GMFM: Gross Motor Function Measure

**Fig. 1:**
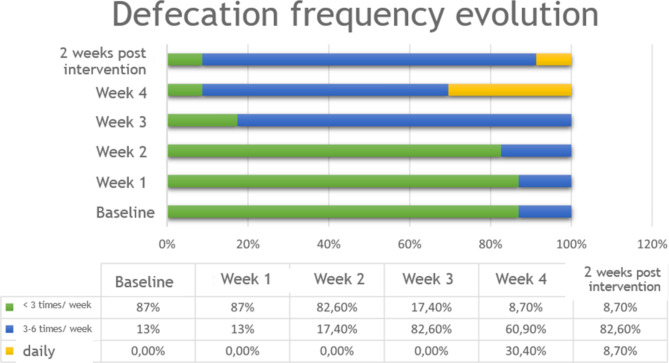
Evolution of the stool frequency

The registry that measured the evolution in defecation frequency, indicated that from 87% (20) who presented a frequency of less than 3 times per week at a basal level, it decreased to an 8.7% (2) after the fourth week of intervention. In addition to this, it could also be observed after four weeks of intervention, that 60.9% (14) of individuals had a defecation frequency of 3 to 6 times per week, and some even had a daily bowel movement (30.4%). This information can be observed in detail in Fig. 1.

There is not a significant association of the Basal Defecation Frequency before the intervention and after 4 weeks of intervention, with a Kendall Tau-b coefficient = 0.295 (*P* = .181). Nevertheless, a relevant change was detected in the weekly Frequency of Defecation as an increase of frequency was observed. Of 20 cases who had a frequency of less than 3 times per week, 90% (18) started having a frequency of 3 to 6 times per week or even daily.

Regarding the 2 cases who maintained a frequency of less than 3 times per week after the 4 weeks of intervention, both were classified at Level V according to the GMFM and were aged 4 and 16 (both with stool type 1–2 hard). The 16-year-old female used 4 enemas per week.

When analyzing the evolution of children, with regards to Defecation Frequency, between the 4th week of intervention and 2 weeks post-intervention, a Kendall Tau-b coefficient = 0.661 (*P* = .013) was obtained. Change was observed in the group of 7 minors who had a daily bowel movement in the 4th week of intervention. After 2 weeks post-intervention, 5 cases (71.4%) now have a frequency of defecation of 3 to 6 times per week. These cases correspond to: 1 female aged 12 with a classification of Level IV and whose stools changed from type 3–5 normal (4th week) to type 1–2 hard (after 2 weeks post-intervention); 2 Level III; 1 Level II and 1 Level I.

**Fig. 2:**
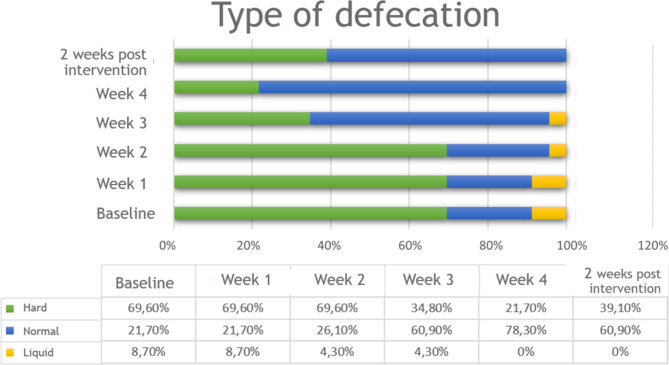
Evolution of the stool consistency

Approximate results were observed when interpreting the evolution of the type of defecation through the BSS. 16 of the participants (69.6%) presented a hard stool at a basal stage and after 4 weeks of intervention it decreased to 5 participants (21.7%). Stools considered normal changed from a 21.7% registered at a basal stage to a 78.3% of participants in the 4th week of intervention. This information can be observed in further detail in Fig. 2.

The relation between the Type of Basal Stool before the application of NESA and after 4 weeks of intervention does not seem to be significant with a Kendall Tau-b coefficient = -0.334 (*P* = .150). However, there seems to be a relevant change in the Type of Stool after applying NESA. Of 16 cases with a Type of Stool 1–2 Hard, on a basal state, 87.5% (14) changed to a Type of Stool 3–5 Normal.

When analyzing the evolution of children, in relation to the Type of Stool between the 4th week of intervention and 2 weeks post-intervention, a Kendall Tau-b coefficient = 0.657 (*P* = .001) was obtained. The change can be observed in the group of 18 minors with a type of stool 3–5 Normal in the 4th week of the application of NESA. After 2 weeks post-intervention, 4 cases (22.2%) changed to Type of Stool 1–2 hard.

With regard to the 5 cases who maintain a Type of Stool 1–2 Hard, they correspond to: 1 female Level V aged 16, 3 minors Level I aged 5 and 7, 1 Level II aged 6.

The average hours of sleep of participants, registered in mean values and standard deviation, could be observed to be 7.35 (0.83) in the basal registry, increasing to 9.09 (1.35) after the 4th week of intervention, and 8.17 (0.7) at 2 weeks post-intervention.

When comparing the hours of sleep between the basal stage and on the 4th week of NESA application, there is not a significant association (Pearson Correlation Coefficient = -0.069; *P* = .755). However, between the 4th week of intervention and 2 weeks post-intervention, a significant association is observed (Pearson Correlation Coefficient = 0.767; *P* < .001).

Regarding the number of interruptions recorder in the sleep diary, a 3.83 (1.95) of average interruptions were observed at a basal stage, decreasing to 1.17 (1.11) on the fourth week of intervention and stabilizing 2 weeks post-intervention in 1.61 (1.34) sleep interruptions.

**Fig. 3:**
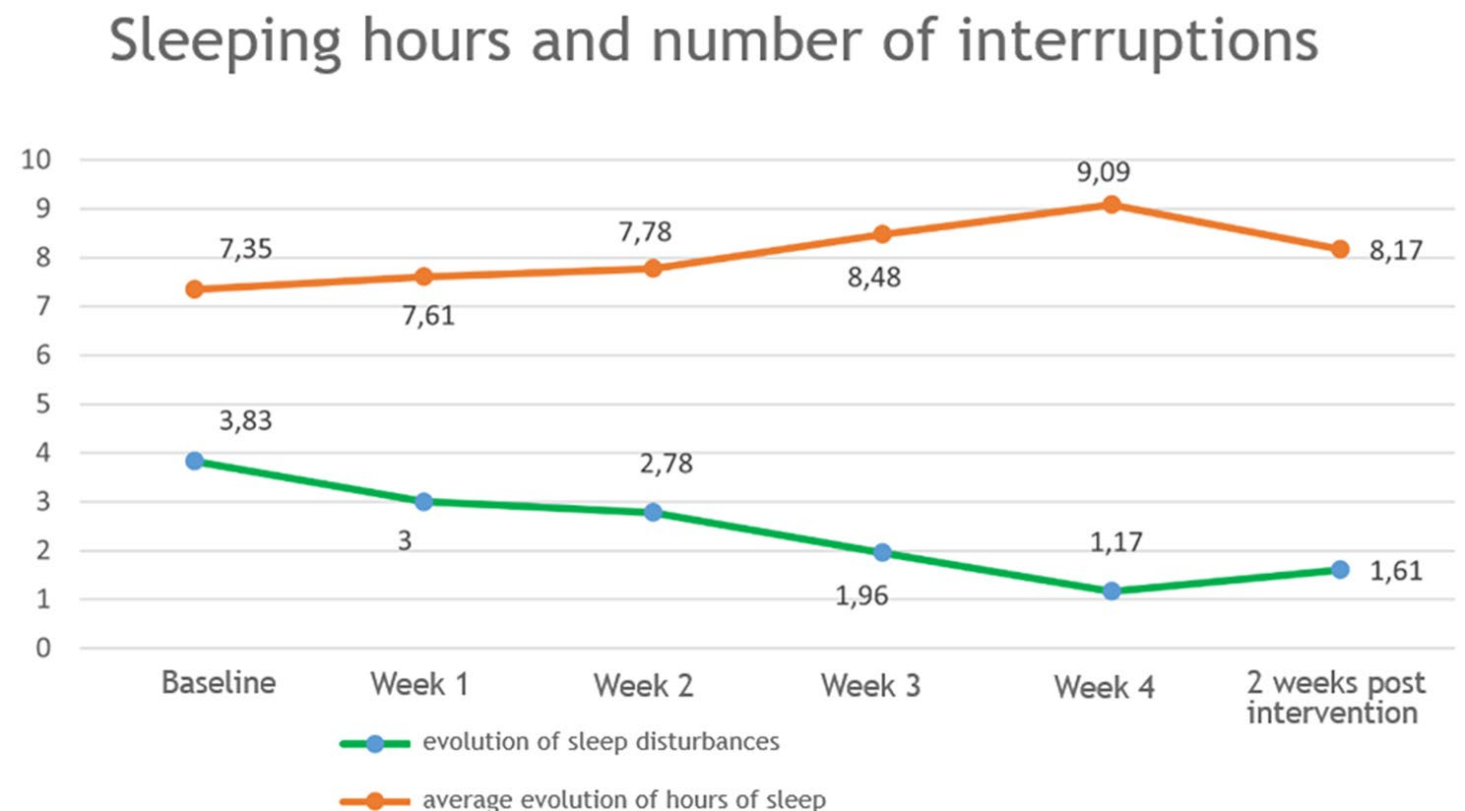
Evolution on sleep characteristics

The evolution of the data registered for the hours of sleep and number of interruptions are represented in Fig. 3.

When comparing the number of sleep interruptions or arousals during the night, it is observed that between the basal stage and the 4th week of NESA application, there is not a significant association (Pearson Correlation Coefficient = 0.224; *P* = .304). When comparing the variable between the 4th week of NESA application and 2 weeks post-intervention, a significant association is observed (Pearson Correlation Coefficient = 0.901; *P* < .001).

## Discussion

The main findings of this study, are based on a sample integrated by 23 minors, aged between 2 and 16 (78.2% are 8 years old or younger and 21.7% are older than 8), the majority are female (73.9%) and with a Level of III or below according to the GMFM (86.9%). We must emphasize that there were no adverse events in 100% of cases during the study and no incidences were registered. In the descriptive analysis of data, relevant improvements were detected in relation with the Frequency of Defecation, Type of Stool, number of hours of sleep and the number of interruptions occurred during sleep. It can also be observed that 91.3% of cases did not require enemas during the time the study was conducted.

An essential aim of the study was to ensure safety of participants in the application of microcurrents. The applied level of intensity of microcurrents in the present study, was significantly below the sensation threshold of children, therefore participants did not experience anything during the application of microcurrents. As expected, and similarly to previous studies, there were no adverse effects or events, as the stimulation with microcurrents is done in microamperes, which mimics the electrical intensity found on live tissue. [20–22] Nevertheless, it must be highlighted, that the present study is the first one that evaluates its effects on constipation and quality of sleep in children with neurodevelopmental disorders. With regard to healthy children, there is only one article registered where only one participant was treated. [22].

Regarding safety and its relation to the application of microcurrents in the treatment of children of a similar age group, some studies have established clinically relevant results in the application of low frequency electrotherapy and its influence on urinary incontinence problems or in overactive bladder. [23–25] Other previous studies demonstrate good results and lack of complications using non-invasive neuromodulation in fecal incontinence, however, the methodology used was completely different as most of them are based on the stimulation with neuromodulation of the posterior tibial nerve or of the sacral plexus. [26, 27] Other important factor to bear in mind is the characteristics of participants, as up to the present, interventions have been carried out on children without added or associated pathologies.

Family members and professionals who attended to the children in the Centre of the study, reported that they had observed a lesser effort of the child when defecating. From a physiopathologic perspective, pain during defecation has traditionally been considered an important etiological and perpetual factor of constipation physiopathology. Pain or overexertion during defecation can induce a feces retention behavior. [28].

Feces with a retention behavior can lead to fecal impaction with the presence of a large fecal mass in the rectum and the inability to pass stool. [29] In contrast with the previously mentioned studies, most participants could not verbally express the level of pain of effort due to their disability level, so it was based on observing their expressions and communication during defecation.

There is agreement with other research in that parents had assessed the consistency of the feces based on the memory of the feces of their child. This is very relevant in clinical practice, as parents are usually the ones who report the consistency of the feces of their child, both, in clinical settings and in clinical trials. [15] In addition, the analysis methods performed in the detection of the type of feces was similar to previous studies. [30–32].

Regarding the evaluation of the quality of sleep, even though polysomnograpy is considered the Gold Standard, previous studies indicate that a sleep diary is within acceptable limits for all sleep parameters, and that the differences between a sleep diary and actigraphy, are acceptable for the beginning of sleep and wake time. [33, 34].

If the focus is on the management of sleeping disorders in children with neurodevelopmental problems, evidence is limited due to small sample sizes, observational studies without a control group, lack of objective measures results and lack of generalizability. [35] Up to the present, sleep hygiene has been the therapy of first choice [36, 37] and pharmacological treatment as a complementary therapy with the aim of minimizing sleep alterations in children. The role of medication as a treatment for sleep problems in these children, remains controversial. Many studies have indicated that using hypnotics and sedatives in the treatment of sleep disorders in children is a very common practice. [38–40] It was observed that in all cases, there is a heterogeneity in the clinical presentation of the type of population. In our study, in order to ensure the possible influence of non-invasive neuromodulation in sleep, no sleeping habits in relation with sleep hygiene or pharmacological treatment were modified.

The current study must recognise some limitations. In the first place, it was a cross-sectional study, therefore, it did not allow any definitive causal inference. In a near future, it would be necessary to develop a randomized controlled trial, even with the possibility of narrowing down to a specific disability level according to the GMFM. In the second place, influence could not be determined of different structural and postural underlying pathologies, as a thorough assessment of imaging studies was not performed. In the third place, the number of subjects was small and further research is needed with a larger population to evaluate the usefulness of microcurrents as a treatment to improve constipation and quality of sleep in children with neurodevelopmental disorders. In the fourth place, considering that this study was carried out in a relatively short period of time, although no-treatment effects were measured two weeks post intervention, additional studies must be performed to evaluate long term effects.

## Conclusions

The Non-invasive neuromodulation has resulted to be a safe technique, easy to use and with clinically relevant results, it is suggested to widen lines of research that allow us to develop randomized controlled trials with larger samples, including a multicentric type intervention, and where an assessment of the quality of life of the child and their families could be added.

## Data Availability

All data generated or analysed during this study are included in this article.

## Abbreviations

ADHD: Attention deficit/hyperactivity disorder
BSS: Bristol Stool Form Scale
GMFM: Gross Motor Function Measure
NESA: Non-invasive Superficial Neuromodulation
PLMS: Periodic limb movements in sleep
RLS: Restless leg syndrome
SPSS: Statistical Package for the Social Sciences
STROBE: Strengthening the Reporting of Observational Studies in Epidemiology
